# Flow With Nature Treatment for Depression: Participants’ Experiences

**DOI:** 10.3389/fpsyg.2021.768372

**Published:** 2022-01-05

**Authors:** Kirsi Salonen, Katriina Hyvönen, Jane-Veera Paakkolanvaara, Kalevi Korpela

**Affiliations:** ^1^Faculty of Social Sciences/Psychology, Tampere University, Tampere, Finland; ^2^Institute of Rehabilitation, JAMK University of Applied Sciences, Jyväskylä, Finland; ^3^Department of Psychology, Faculty of Education and Psychology, University of Jyväskylä, Jyväskylä, Finland

**Keywords:** nature-based intervention, nature-based rehabilitation, comprehensive nature experiences, connectedness with nature, flow with nature treatment

## Abstract

This study examined Flow with Nature (FWN) treatment, which is an integrative intervention (rehabilitation) based on eco and environmental psychology, psychotherapeutic theories and professional psychological practice. FWN is intended for depression rehabilitation with the help of social support, nature environments and FWN exercises. Exercises encourage sensing the environment, mindful awareness, psychological processing and focusing on the future. The FWN treatment proceeds in separate stages (horizon, growth and path), which emphasise nature, group (social support) and FWN exercises differently. This study focused on the experiences of the participants in the FWN treatment. Finnish adults who had been diagnosed with clinical depression took part in the FWN treatment (*N* = 82) and answered feedback questions (by paper, electronic questionnaire or phone discussion). Answers were analysed using theory-based content analysis. Data were collected between spring 2019 and spring 2020. The majority of the participants were women (82%) and on average 44 years old. Content analysis revealed that the participant feedback answers were in agreement with the central theoretical themes of FWN. The participants emphasised the significance of nature, social support and exercises differently. Moreover, the significance of these ingredients differed according to the stages of treatment: in the horizon stage restorative (e.g., fascination) and comprehensive nature experiences (e.g., connectedness with nature), in the growth stage social support (e.g., peer support) and in the path stage environmental self-regulation (e.g., nature as a part of life) were emphasised. These results are in accordance with the objectives of the stages and seem to support the phase-based rationale. The participants’ experiences of the key elements of the intervention, social support and nature environment were experienced mainly positively, which supports their inclusion in future intervention versions. In the future, FWN exercises should be developed to better enable participants’ possibilities for concentration and being present in the moment (mindfulness). Exercises should also be simplified to match the target group’s cognitive abilities.

## Introduction

There is a need to develop new and effective treatments for stress-related mental disorders (e.g., depression) that reduce individual suffering as well as its high costs to society. Nature experiences have been linked to improved self-esteem among people suffering from mental illness (Barton et al., 2012), and nature-based interventions (NBIs) have shown positive effects in the treatment of and rehabilitation from stress-related illnesses, such as depression (Kim et al., 2009; Annerstedt and Währborg, 2011; Sahlin et al., 2012, 2015; Währborg et al., 2014). For example, nature settings seem to strengthen the benefits of cognitive behavioural therapy for major depressive disorder (Kim et al., 2009). Using nature walks as a part of the ‘Coping with Depression’ program resulted in a significant decrease in depression and an increase in positive mental well-being over an 8-week intervention and a 3-month follow-up (Korpela et al., 2016). In a series of single-group studies of a 12-week therapeutic horticulture programme with clinically depressed adults, a significant change for the better was found in several mental health variables, such as depression and anxiety, during the intervention (Gonzalez et al., 2011).

A meta-analysis of relatively long-term NBIs (5 weeks, on average) including adults with or without mental and/or physical health problems revealed that NBIs were effective for improving positive affect and reducing anxiety, depressive mood and negative affect (Coventry et al., 2021). The most effective interventions lasted between 8 and 12 weeks, and the optimal duration ranged from 20 to 90 min. Another review of short-term NBIs (from 1 min to 2 days in duration) for adults indicated that they improve positive mood, decrease negative mood, stress and anxiety and increase physical activity (Wilkie and Davinson, 2021). In these studies, ‘nature-based’ refers to time spent in both unmanaged nature and publicly accessible, managed parks and gardens. However, the majority of such NBI studies lacked proper descriptions of the settings and intervention techniques as well as theoretical frameworks guiding NBI designs (Wilkie and Davinson, 2021). In our study, we have given consideration to these aspects of the study design.

We focused on nature-based rehabilitation for depression, which is a psychiatric syndrome with several symptoms: low mood, loss of interest or pleasure, fatigue, decrease in self-confidence, feelings of worthlessness, excessive feelings of guilt and self-blame, thoughts about suicide or self-harm, self-harming behaviour, decreased concentration, either slowed or agitated movement, sleep disturbances and changes in appetite or weight (ICD-10 diagnostic system; Finnish Current Care Guidelines, 2016).

*Flow with Nature* treatment (FWN) is a theory-, research- and practice-based rehabilitation intervention for clinical depression. The aim is to reduce depression symptoms and to support well-being and psychological processing. FWN was developed based on a previous 5-week NBI (*FWN group*; Salonen et al., 2018, 2020; Salonen and Törnroos, 2019; Salonen, 2020) for promoting occupational well-being. The results of that 5-week intervention showed, first, that attending the group promoted visiting and experiencing nature in all seasons. Second, comprehensive nature experience (e.g., connectedness with nature; Salonen et al., 2016) and awareness of perceived impacts of nature were strengthened and participants could process problematic psychological issues with the help of nature elements. Third, negative nature experiences were reduced.

### Flow With Nature Treatment

The FWN treatment is an integrative intervention (rehabilitation) based on theories of restorative environments (Attention Restoration Theory: Kaplan and Kaplan, 1989; Kaplan, 1995; and stress reduction theory: Ulrich, 1983) and so-called comprehensive views on human–nature relation, such as environmental self-regulation (favourite place studies: Korpela and Ylén, 2009), nature connectedness (e.g., CNS: Mayer et al., 2009), deep reflections in nature (Winter and Koger, 2004; Brymer et al., 2010) and comprehensive nature experience (Salonen et al., 2016; Salonen, 2020). Furthermore, this intervention includes elements from Mindfulness-Based Cognitive Therapy (e.g., Hayes et al., 2012) and the Transtheoretical Model of Behaviour Change (Prochaska et al., 2013). The intervention is strongly rooted in the first author’s years of professional psychological practice involving nature-based methods in therapeutic work with clients suffering from mental disorders. The developers of the FWN methods are researchers but also professional psychologists and psychotherapists.

The FWN treatment is based on the support of both social interaction (social support) and natural environments (environmental self-regulation). The following general principles are characteristic to FWN: regular social support (e.g., weekly meetings), psychological and physical safety (e.g., support from the facilitator and group, safe environment), respect for other group members as well as nature (i.e., everyone’s experiences and favourite places are respected), flexibility (e.g., the FWN exercises are optional and the participants’ acute needs are primary) and responsibility (i.e., for rehabilitation and towards the environment).

In addition to social support, FWN participants are encouraged to recognise nature environments’ significance for one’s own well-being, and they receive support for psychological and environmental self-regulation. Environmental self-regulation refers to the use of physical settings and their experiential contents as a means of self-, affect- and stress-regulation (Korpela et al., 2018). Nature is not only a physical stage in which the rehabilitation takes place, but an essential part of the rehabilitation. For example, the participants are instructed to experience nature with many senses and be mindfully aware of their surroundings. In addition, supporting nature connectedness is an integral part of the treatment because when individuals experience sameness and continuity with nature they can more easily identify nature elements that reflect and symbolise their experiences and, in turn, help in processing those experiences. In addition, experiences are shared with one another by visiting others’ favourite places and with the help of nature elements.

The FWN treatment has separate stages (Horizon, Growth and Path) that emphasise nature, group (social support) and FWN exercises differently (Table 1). At each stage, there are four different FWN exercises with similar structures: first, experiencing nature individually and then sharing the nature place experiences in pairs or with the group. However, FWN exercises differ from each other according to the contents and emphasis of the stages.

**Table 1:** Separate stages of the Flow with Nature (FWN) treatment.

FWN treatment	Horizon	Growth	Path
Nature-based treatment intervention for depressive patients, supporting mental well-being (also using online meetings).	Shifts attention from inside to here and now and the natural environment.	Supports positive psychological processing.	Focus is on the future time frame.
**Nature**	**Horizon/Nature**	**Growth/Nature**	**Path/Nature**
Natural environment and nature elements (greenery, trees, water, stones, animals, etc.) are relevant.	Recognising favourite places and ways of experiencing nature, becoming aware of the impacts and support of nature (restoration and comprehensive nature experiences).	Recognising possibilities to process negative and unfinished experiences with the support of nature elements and connectedness with nature (e.g., nature symbols).	Recognising possibilities, means and situations in which nature supports mental well-being in the future and in everyday life. Nature experiences (also memories) continue to support the person.
**Group/social support**	**Horizon/Group**	**Growth/Group**	**Path/Group**
Human relations and social support (group and facilitator) are essential.	Recognising a suitable and safe way to be in the group and to gain social support. In this stage, social support with empathy, acceptance and safety (also facilitated) are important. Expressing place experiences through words enhances awareness.	Developing confidence to process and share more deeply within the group and to become aware of the significance of social support (peer support).	Recognising meaning and a need for social support and involvement. Group experiences (memories) continue to support the person.
**FWN exercises**	**Horizon/Exercises**	**Growth/Exercises**	**Path/Exercises**
FWN exercises support the content of Horizon, Growth and Path with the help of nature and human relations.	Exercises focus on grouping, recognising favourite places and supporting nature connectedness.	Exercises support psychological processing	Exercises (and homework) direct the attention to the future.

In the first stage, Horizon, the focus is on shifting attention to here and now with the help of a natural environment (‘The mind opens to the horizon’). This mindfulness makes it possible to be present in the moment and in the group in a safe and permissive way. The Horizon stage is largely based on the triphasic *favourite place* exercise (Salonen, 2012), where mindfulness in nature and nature connectedness are supported in three different phases (individual, couple, group) with the help of favourite place exercises and by sharing the nature place experiences. The Horizon exercises focus especially on grouping (e.g., visiting each other’s favourite places), recognising favourite places and supporting nature connectedness (e.g., *What do I need*? exercise). At the Horizon stage, the participants can, for example, recognise favourite places in which nature impacts them most positively and significantly.

In the second stage, Growth, the focus is on supporting mental growth and positive psychological processing, such as psychological flexibility, positive change in self-perception and personal emancipation. The growth exercises focus on processing the history of one’s life (*Life cycle* exercise), negative experiences (*Problem place* exercise), psychological flexibility (*Visit emotive places* exercise) and positive self-image (*Nature feedback* exercise). At the Growth stage, the participants become more aware of natural elements that reflect one’s own feelings or unfinished experiences (symbols), and they acquire tools for psychological processing with the help of nature symbols and social support (e.g., peer support).

The final stage, Path, focuses on the future time frame (‘The path takes you forward’): experimenting with alternatives and affirming positive change (Prochaska et al., 2013) in everyday life after FWN. The Path exercises include allowing dreams about a desirable future (*Nature dream map* exercise), experimenting with change alternatives (*Towards change* and *Nature anchor* exercise) and supporting positive self-perception (*Nature present* exercise) as well as environmental self-regulation (Korpela and Ylén, 2009). At the Path stage, the participants are supported in engaging themselves in future outdoor recreation and in using FWN exercises both during meetings and after rehabilitation (‘FWN independent’). ‘FWN independent’ exercises are developed for participants’ independent use (without the group) and are shared by e-mail.

The present study focuses on the experiences and feedback of the FWN treatment participants here also refers to RCT research. Our first aim was to present the central theoretical views, objectives and methods of the FWN treatment, and, secondly, to qualitatively describe the occurrence of the central theoretical themes in the FWN treatment participants’ feedback.

## Materials and Methods

### Data Collection and Participants

The present substudy is part of a larger intervention study (funded by the Finnish Social Insurance Institution) on the effectiveness of nature-based treatment in groups for treating depression in which the data were collected through RCTs. In the RCT study, the participants were randomly allocated to either a treatment group (FWN added to TAU) or control group (TAU). The treatment groups were carried out between the spring of 2019 and spring 2020. In addition, the participants in the control group had the opportunity to attend the treatment groups *after* their follow-up period. The present research focuses on the data (*N* = 82) collected from participants who took part in the group treatment and answered feedback questions after the intervention period (by paper, electronic questionnaire or phone discussion). The majority of the participants were women (82%), and they were on average 44 years old.

Flow with nature treatment meetings took place in five cities across Finland and there were altogether 16 such groups. Every group had 12 meetings held in nearby nature like parks, urban and rural forests and by water. Each meeting lasted one-and-a-half hours. The FWN group included 3 to 10 participants (around 6 on average). The groups were supervised by health care and social services professionals (psychologists and an occupational health nurse) who had received training for the FWN treatment. During restrictions caused by the Covid pandemic in the spring of 2020, the FWN treatment was conducted *via* online meetings so that each participant was in their own favourite place in nearby nature, and the experiences were shared *via* video call (for example, using a smartphone).

The feedback form included questions on experiences during the FWN treatment: *What felt important?* (i.e., in the group, nature and exercises); *What felt difficult?* (i.e., in the group, nature and exercises); *Was there something unnecessary?* (i.e., in the group, nature and exercises); *What are your memories of your nature experience?*; and *Any other comments?* Answers were analysed using theory-based content analysis (Tuomi and Sarajärvi, 2002) and a model or a categorisation frame (FWN model) that was developed for this study (see Table 2). In the analysis of the data, the written descriptions were read through and theoretical coding (without the computer programme) was used for the categorising. Similar expressions were grouped together to form a category. In the theory-based content analysis, main categories and subcategories were examined in relation to the FWN model. In other words, the participants’ experiences (both positive and negative) were categorised in relation to the FWN treatment and the separate stages (Horizon, Growth, Path). The FWN model was used as a framework when identifying the themes.

**Table 2:** Flow with nature model (a categorisation frame).

FWN treatment	Horizon	Growth	Path
Description of the FWN treatment in a way that separates stages is not emphasised.	Descriptions that emphasise multi-sensory sensing of the environment, nature connectedness and mindful awareness.	Descriptions of psychological processing are accentuated.	Descriptions focusing on the future are highlighted.
**FWN in general**	**Horizon stage in general**	**Growth stage in general**	**Path stage in general**
Descriptions of the whole FWN treatment (also with online meetings) in general (positive and negative).	Descriptions of the content of the Horizon stage in general (positive and negative).	Descriptions of the content of the Growth stage in general (positive and negative).	Descriptions of the content of the Path stage in general (positive and negative).
**Nature**	**Horizon/Nature**	**Growth/Nature**	**Path/Nature**
Descriptions of the nature environment and nature elements in general (positive and negative).	Descriptions of the Horizon stage in a way that recognition, awareness and/or support of nature are highlighted.	Descriptions of the Growth stage in a way that recognition, awareness and/or support of nature are emphasised.	Descriptions of the Path stage in a way that recognition, awareness and/or support of nature are accentuated.
**Group/social support**	**Horizon/Group**	**Growth/Group**	**Path/Group**
Descriptions of the social support in general (positive and negative).	Descriptions of the Horizon stage in a way that recognition, awareness and/or support of human relations are highlighted.	Descriptions of the Growth stage in a way that recognition, awareness and/or support of human relations are accentuated.	Descriptions of the Path stage in a way that recognition, awareness and/or support of human relations are pointed out.
**FWN exercises**	**Horizon/Exercises**	**Growth/Exercises**	**Path/Exercises**
Descriptions of the FWN exercises in general (positive and negative).	Descriptions of the Horizon stage in a way that recognition, awareness and/or support of FWN exercises are emphasised.	Descriptions of the Growth stage in a way that recognition, awareness and/or support of FWN exercises are highlighted.	Descriptions of the Path stage in a way that recognition, awareness and/or support of FWN exercises are accentuated.

To assess the reliability of the coding, an independent second coder analysed 50% of the data on the basis of the FWN model in the same way as the first coder. The evaluation compatibility was 84%.

## Results

The content analysis of the feedback questions revealed four main categories: FWN experiences, Horizon experiences, Growth experiences and Path experiences. Every main category had four subcategories in which descriptions in general, nature relatedness, human relations (social support) and FWN exercises varied. The central theoretical themes (separate stages) of FWN were found in the participants’ feedback, although there were less descriptions of Horizon, Growth and Path experiences than general descriptions of FWN experiences (without descriptions of the stages).

### Flow With Nature Experiences

Feedback on the FWN treatment included descriptions of the group in general as well as descriptions of the significance of the natural environment, social support and FWN exercises (without descriptions of the separate stages of the treatment).

The participants appreciated regularity, routines, peacefulness, freedom and flexibility. It seemed to be important that the whole treatment was based around the natural environment. FWN was also thought to have the potential to support one’s own responsibility in rehabilitation or changing their own participants’ conception of depression:

42 (participant code): *In general, participation in FWN provides the possibility for individuals to use the whole [FWN] process for the improvement of their own health and emphasises everyone’s own responsibility for recovering […]. The therapy provides an extremely good setting for this, for insights and contemplating issues. One must only let go and experience the process of FWN*.

30: *The group [FWN treatment] also succeeded in challenging my assumptions regarding depression*.

Negative experiences of the FWN related to changes in the schedule or negotiating meeting places together. There were also desires to attend to other illnesses and/or psychological conditions (e.g., migraine and autism spectrum).

Negative descriptions of participation were also connected to leaving home (to attend meetings) and completing the study questionnaires (before and after meetings). There were also negative and positive experiences reported in the same description:

51: *The most difficult issue was to go there, but after that I had a “Yes!” feeling that I came*.

Participants felt both positive and negative perceived impacts. The latter one was, for example, the difficulty of changing a negative self-concept. Participants positively felt that FWN empowered them, strengthened their feelings of gratitude and improved their mood. FWN was compared with another Finnish rehabilitation activity (called Silmu):

69: *I got more from this than I got from the Silmu rehabilitation*.

The online connections were experienced both negatively and positively:

3: *Actually, meeting via online connection helped me to enjoy nature alone as well*.

The significance of nature in FWN in general comprised descriptions of both peaceful and disturbing factors in nature (e.g., bad weather, dark seasons, littering, disturbance from traffic, mechanical sounds, urban nature or other people), where peace in nature is defined as the peaceful characteristics of the natural environment and possibilities for oneself to be ‘at peace’ in nature:

77: *Personally, I experienced the best moments in peaceful, quiet places irrespective of what kind of a natural place it was*.

The significance of the social group in FWN in general consisted of descriptions of positive experiences of social support and negative experiences of social situations. The participants appreciated peer support, small group size, confidential atmosphere, open discussion, accepting differences and having an empathic group facilitator. Empathy among the group and gratitude for the empathic group facilitator were described as follows:

23: *It was as if I had a new family*.

15: *You [facilitator] are so hearty and genuine and can empathise so well with others’ feelings! You listen intently and comment wisely. You take everyone into consideration really well*.

The group situations may also have been strained and loaded (negative experiences of social situations), and it may have been difficult to verbalise one’s experiences and share experiences with another group member. There was one negative feedback point for a facilitator because of the lack of ‘warmth’. There was also one instance when a facilitator went to talk with a participant in her favourite place and the other participants could not see her for a while (even though they were quite nearby). The situation seemed stressful to one participant.

The significance of nature in FWN in general related to descriptions of the flexibility and difficulty of the FWN exercises. The participants may have experienced the exercises as proposals and as possibilities that could be flexibly edited per individual, but there was also feedback suggesting that some exercises were seen as too strictly ordered and that activities were to be done even if they felt too difficult or unsuitable. This is an example of the flexibility of an FWN exercise:

10: *There was no exact template for the exercises, but I was able to carry them out. It felt natural to me in that moment*.

### Horizon Experiences

The second main category was Horizon experiences, which included descriptions from the Horizon stage in general and descriptions about the significance of the natural environment, social support and FWN exercises.

Descriptions of Horizon experiences in general contained feedback on the contents and objectives of the Horizon stage without quotations about nature, human relations or exercises. The positive descriptions were connected to mindfulness:

34: *Becoming quiet, calming down, listening to oneself and hearing*.

There were also difficulties with mindfulness, at least at the beginning of the meetings or when coming from work.

The significance of nature in the Horizon stage included descriptions of restoration (e.g., fascination), comprehensive nature experiences (e.g., connectedness with nature or feelings of being accepted) and obstacles (e.g., difficult to become part of nature). For example, in the following quote the participants felt accepted by nature and experienced a sense of nature connectedness:

74: *[It felt good] to hug trees and to lean on them, and to sense the acceptance of nature toward me*.

50: *Not everybody has a deep nature connection, but it can be learned in nature*.

The significance of social support in the Horizon stage was seen mainly positively: grouping, sharing place experiences; but also reluctance to share one’s place experiences. These examples represent grouping and sharing place experiences:

63: *It is important to become an equal member of the group*.

55: *It was important that I heard the comments of other group members regarding my own findings [favourite places and nature elements]*.

The participants appreciated that with the help of the Horizon exercises it was possible to rehearse mindfulness in nature, even though concentrating on the exercises was sometimes difficult:

75: *It’s a suitable way to be in nature; the favourite place exercise [mindfulness exercise] was good and important because there is always something [disturbing] in progress in the mind*.

63: *It was always possible to experience nature like in a favourite place exercise*.

29: *I noticed that sometimes it was more difficult to concentrate on the exercises, but I accepted it for myself and so it did not really disturb me*.

### Growth Experiences

The third main category, Growth experiences, included descriptions of perceived impacts of psychological processing of inner experiences, which in some cases included both positive and negative aspects. Inner experiences were described with the help of nature symbols.

53: *Even though difficult issues were dealt with, it [FWN] still lightened my feelings*.

57: *And I cannot identify feelings or emotional states, so we [participants] had to think about them really*.

57: *I remember the wonderful dead wood that greatly reflected my own instability*.

21: *I remember the stone that had split and the image that it created, [it was] symbolic. Even an extremely strong one can break in two down the middle yet still be beautiful and continue life split yet strong*.

The significance of the group in the Growth stage included descriptions of positive and negative (e.g., obstacles to confidence) experiences in the therapy group. The following example shows what a participant felt was important (positive):

63: *[I felt] strong confidence that I could talk about my own issues in the group. The experience of peer support made me realise that I was not alone with my problems […] and that others have difficulties solving such problems as well*.

The exercises in the Growth stage focused on psychological processing (support for processing) as can be seen in the following comment during the ‘Life cycle’ exercise:

27: *The life paths that relate to childhood or the past can also be found in a forest: For example, that pile of brushwood looks like my teenage years, and that clear path along a few rootstocks that are easily crossed looks like my “young adult’s uncomplicated working life”. Those small, beautiful spruces brought my father to my mind*.

There were also difficulties in benefitting from the exercises because they were felt to be unsuitable by some participants. For example, finding metaphors (nature symbols) was difficult or the theme not interesting for some. In those cases, difficulties may have been there at the beginning, but the perception of nature may have eventually eased this processing, like the following quote shows:

23: *The most impressive experience was perhaps in the meeting in which the task was to remember the past and identify what kind of thoughts arise. The topic did not quite convince me, but when walking in the forest I paid attention to the stubs that the moss had covered. Then I kept looking at them in more detail and was surprised at what message they gave*.

### Path Experiences

The fourth main category, Path experiences, included descriptions of new behavioural patterns and difficulties in thinking about the future. New behavioural patterns were indicated, for instance:

15: *In my opinion, the important matters were experimenting with and practising the new behavioural patterns*.

The significance of nature (nature as a part of life) in the Path stage was seen as a stronger nature connection, increased outdoor recreation or environmental self-regulation. Also, elements or memories related to nature were described as having stayed in mind and were experienced concretely as favourite places, as the following comments show:

46: *I rediscovered the small nature reserve with its small lakes near my home. Nearly every day and in every weather, I circled the lake and enjoyed the scenery […]. Every time, I returned home with a calm mind*.

38: *During the last group session, I found a hut formed by two decorated birches. I thought that it looked like the kind of home we might like to move into in the future. The hut was sheltered and beautiful, and I hoped that there would be big trees in the yard of our future home and a forest nearby because nature supports my well-being*.

18: *I have often returned there [favourite place that was discovered in a group meeting]*.

Memories of the group experiences or shared favourite places (group experiences as a resource) were experienced as important:

22: *The story of the caves [told by another participant] has stayed in my mind in particular and always comes to mind when there is a hole or a pit or some sort of cave in the ground*.

In addition, some participants voluntarily wanted to keep in touch with the other group members; some formed a messaging group and others resolved to take nature walks together.

The participants felt that at least parts of the exercises could be used after rehabilitation as well (exercises like *Nature anchor*). The following participant felt that the exercises could be used during depressive moods:

41: *I know that I am able to use some exercises even if I’m depressed, if only the [external] conditions are suitable for my mental needs*.

Some participants particularly pointed out the Path exercise (complex exercise) from the eleventh meeting (‘Towards change’ exercise) because it was felt to be too complicated. There was also a suggestion that all exercises for independent use (FWN independent) should be sent by e-mail to all participants after every group meeting.

### How Often Different Themes Were Mentioned

Table 3 shows that FWN experiences (60.3% of all participants) were mentioned more often than others and received the highest number (39%) of positive comments. In the subcategories, FWN in general (positive experiences of FWN content, participation, perceived impacts and online meetings, 13.3%) had the highest number of positive mentions, but also the significance of social support (positive experience of social support, 12.6%) as well as the significance of nature (peaceful places in nature, 8.9%) had moderately high percentages. In the subcategory of the significance of FWN exercises, there were more negative experiences (e.g., difficulty of FWN exercises) than positive ones (flexibility of FWN exercises).

**Table 3:** Participants’ experiences in the *FWN* treatment (main categories and subcategories; frequency and percentage of mentions in the main categories).

***Flow with Nature* experiences (368) 60.3%** (positive 39%)	**Horizon experiences (125) 20.5%** (positive 13.5%)	**Growth experiences (64) 10.5%** (positive 9.2%)	**Path experiences (53) 8.7%** (positive 6.9%)
FWN in general: Positive experiences of FWN content, participation, perceived impacts and the online meetings **(81) 13.3%** Negative experiences of FWN content, participation, perceived impacts and the online meetings **(33) 5.4%**	Horizon stage in general: Mindfulness **(12) 2.0%** Difficulty with mindfulness **(15) 2.5%**	Growth stage in general: Perceived impacts of psychological processing **(8) 1.3%**	Path stage in general: New behavioural pattern **(4) 0.7%** Difficulties with thinking about the future **(1) 0.2%**
Significance of natural environment in general: Peaceful places in nature **(54) 8.9%** Disturbing factors **(23) 3.8%**	Nature in Horizon stage: Restoration/comprehensive nature experience **(45) 7.4%** Obstacles to restoration/comprehensive nature experience **(7) 1.1%**	Nature in Growth stage: Nature symbols **(9) 1.5%**	Nature in Path stage: Nature as a part of life **(26) 4.3%**
Significance of social experiences: Positive experience of social support **(77) 12.6%** Negative experiences of social situations **(38) 6.2%**	Human relations in Horizon stage: Positive experiences of grouping, sharing place experiences **(14) 2.3%** Negative experiences of grouping, sharing place experiences, reluctance to share **(5) 0.8%**	Human relations in Growth stage: Experience of therapy group **(31) 5.4%** Obstacles to confidence **(4) 0.7%**	Human relations in Path stage: Group experiences as resource **(5) 0.8%**
Significance of FWN exercises: Flexibility of FWN exercises **(27) 4.4%** Difficulty of FWN exercises **(35) 5.7%**	Exercises in Horizon stage: Rehearsing mindfulness **(11) 1.8%** Difficulties in concentrating **(16) 2.6%**	Exercises in Growth stage: Support from exercise **(8) 1.3%** Inconvenient exercise **(4) 0.7%**	Exercises in Path stage: Exercises that can be useful in future **(11) 1.8%** Complex exercise **(6) 1.0%**

Horizon experiences (20.5%) received the second highest number of mentions, the majority being positive (13.5%). The subcategory of nature at the Horizon stage (restoration/comprehensive nature experience, 7.4%) received most of the positive mentions among the Horizon experiences. In two subcategories (Horizon in general, Horizon exercises), negative experiences (difficulties with mindfulness and concentration) were mentioned slightly more frequently than positive experiences. Growth (10.5%) and Path (8.7%) experiences were mentioned less often, but in both cases the quotations were mostly positive (Growth 9.2% and Path 6.9%). In addition, every subcategory of the Growth and Path stages had more positive mentions than negative ones. In four subcategories (specifically of the Horizon and Growth stages), only positive experiences were mentioned: nature as a part of life (4.3%), nature symbols (1.5%), perceived impacts of psychological processing (1.3%) and group experiences as a resource (0.8%).

When the results are examined as a whole, mentions of social support comprised 28.5% (21% positive), those of nature 27% (22% positive), those of FWN in general 25.2% (including separate stages, 17.2% positive) and those of FWN exercises 19.3% (9.3% positive).

## Discussion

In summary, this study examined the FWN treatment, intended for the rehabilitation of depression with the help of social support, nature environments and FWN exercises. Exercises encourage multi-sensory sensing of the environment, mindful awareness, psychological processing and focusing on the future. These central theoretical themes of the FWN treatment were found in the feedback from the participants in the FWN treatment. It was also observed that the significance of nature, social support and exercises differed at the different stages.

How often the different themes were mentioned reveals that the FWN treatment was experienced mostly positively. FWN experiences, which included positive experiences of social support, peaceful natural environments and FWN in general (e.g., regularity, participation and perceived impact), were mentioned equally often in participants’ feedback. These factors are the ones on which the FWN is based.

Among the positive experiences mentioned at the separate stages, nature and social support were emphasised differently. At the Horizon stage restorative (e.g., fascination) and comprehensive nature experiences (e.g., connectedness with nature) were mentioned most often, at the Growth stage social support (e.g., peer support) and at the Path stage environmental self-regulation (e.g., nature as a part of life). These results are in accordance with the objectives of these separate stages and seem to support the phase-based rationale. In the Horizon stage, the purpose is to make it possible to be present in the moment (mindfulness) in nature, so that restoration and comprehensive nature experiences are facilitated. In the Growth stage, the focus is on psychological processing (also of problematic issues), so the confidential social support is essential. In the Path stage, the idea is that positive nature experiences become part of environmental self-regulation and part of a person’s life.

Negative experiences mostly related to difficulties (participation, concentration, mindfulness, online meetings and FWN exercises). When considering the target group (depressed persons), the results are at quite understandable. For example, having difficulties leaving home, concentrating or being present in the moment (mindfulness), especially at the beginning of the FWN intervention (Horizon stage), may be typical features of depression (ICD-10 diagnostic system; Finnish Current Care Guidelines, 2016). An important part of FWN is that participants are asked to approach these challenges and practise such behaviours. In addition, difficulties with online meetings and FWN exercises should be taken into consideration in the development of future versions so that they do not cause an unnecessarily heavy psychological load.

Frequency results revealed that the natural environment was mostly experienced positively. Disturbing factors in nature related mainly to seasons, weather conditions or disturbances caused by people (e.g., littering and traffic). A previous study suggests that with the help of a NBI, these kinds of negative experiences can be reduced (e.g., Salonen, 2020).

### FWN in General

The results for the perceived impacts of FWN are similar to those of earlier studies on restoration (e.g., Kaplan and Kaplan, 1989) and health experiences (e.g., Salonen and Törnroos, 2019). Feedback about growth experiences also suggests that FWN can support the rehabilitation process, which is in line with earlier studies (e.g., Salonen, 2020).

It was also shown that participants appreciate the general principles of FWN, including regularity, safety, flexibility and freedom. The results give rise to the idea that FWN could increase participants’ own responsibility for rehabilitation and recovery because of the possibilities and tools of FWN for processing one’s issues by oneself. This view is also supported by a study in which the participants in the FWN treatment group experienced their work or study ability to be better at the end of the intervention than did participants in the control group who received only TAU. However, not all participants may be ready for such a responsibility. Participants differed from each other regarding their psychological and physical ability to function. Leaving home was already a big step for some of the participants, whereas others started to utilise the FWN exercises independently from the start.

Participants also differed from each other in regard to their experience of safety. For example, in one group the facilitator spent a short time with one participant in the same area but could not be seen by others. That was felt as distressing by some participants. Consequently, flexibility and freedom may also cause uncertainty and stress. To some participants, exact limits and schedules mean safety. In future, there could be two facilitators per group so that the other facilitator can conduct individual supervision when necessary without causing uncertainty in the minds of other members of the group. New forms of FWN could also be developed for different groups according to their ability to function, experiences of safety or severity of depression symptoms, such as FWN for slight and moderate depression and separately for severe depression. In the latter, more attention could be paid to psychological safety (e.g., two facilitators in a group) and social support (e.g., support leaving home).

According to the current results, participants might re-evaluate their view of depression during the FWN treatment. The reason for this may be that all of the FWN themes (e.g., background views and exercises) consider human experiences common to most people, including: obstacles to achieving restoration or a positive self-concept, difficulties in noticing one’s own needs, unfinished psychic work of past experiences or lack of self-compassion. FWN does not emphasise depression as a disorder even though the treatment is developed for depressed persons; all participants in our study had a depression diagnosis. The participants’ own view of depression and its changes was not separately or specifically asked about, hence this topic would require further study.

### Support From Human and Non-human Nature

Flow with nature consists of three different stages (Horizon, Growth, Path) in which the orientation towards nature, human relations and exercises varies. Despite this, the results are discussed here as a whole so that a more comprehensive and holistic picture of the FWN treatment is obtained.

The FWN treatment is based on the support of the social group and nature elements. The results indicate that the participants complied well with the exercises and were able to use the guidance provided by their facilitator. At the beginning (Horizon stage), the formation of the group was relevant and made possible with the help of the facilitator (e.g., accepting atmosphere) and certain exercises (e.g., the triphasic *favourite place* exercise). After that (Growth stage), stronger confidence within the group became essential so that even difficult issues could be shared and processed. Processing was made possible through growth exercises (e.g., *Life cycle* exercise). The results also indicated that positive group experiences could continue (Path stage) in some ways after the rehabilitation. For example, the mental image of a favourite place introduced by another group member was felt to be impressive and helpful. Some of the members maintained contact with each other after the rehabilitation but this was not originally the purpose of FWN.

The natural environment is not only a physical stage on which FWN is performed but is also an essential part of the rehabilitation and psychological process. Our study shows that the significance of nature can also be seen as a process in which nature connectedness develops through rehabilitation, as did human relations (social support). This objective was gradually achieved by strengthening connectedness with nature and nature mindfulness with the help of exercises (e.g., Horizon stage exercise *What I need*), peaceful nature environments, restoration and comprehensive nature experiences. After that (Growth stage), nature elements ‘came alive’, symbolising one’s inner experiences (nature symbols). When one feels as being a part of nature, then in turn nature becomes a part of them as well; this way, nature elements can more easily symbolise one’s own experiences. Because of the increase of nature connectedness during FWN, it is understandable that the desire for more nature experiences in the future increases as well. In the last stage of the FWN treatment (Path stage), the focus was on the future time frame and especially ‘nature as a part of life’ was essential. Thus, nature can become a part of environmental self-regulation (Korpela and Ylén, 2009) in one’s own life and also support well-being after rehabilitation.

The FWN group sessions were held in nearby nature, such as in parks. According to the results, factors like traffic, mechanical sounds of urban nature (not peaceful) were experienced as disturbing. This brings a challenge to community planning and politics if we are to retain restorative nature areas suitable for interventions near residential environments; being ‘at peace and in quietude’ in nature is essential for FWN.

The results also showed that some of the participants had difficulties with the FWN treatment, their group, the natural environment or the exercises. Difficulties with the FWN and group experiences concerned, for example: leaving home, obstacles to restoration and comprehensive nature experience, sharing one’s personal issues, lack of confidence in the group and experiencing the facilitator negatively. These are matters that should be taken into consideration more thoroughly in the future, especially with depressed persons. One option could be to lengthen the duration of the FWN period. That would provide more time and possibilities for building confidence between the participants and strengthen their connectedness with nature.

Further, the results showed that some of the exercises were experienced as difficult and complex, especially the ‘towards change’ exercise. It is important that the exercises do not cause extra cognitive load and that there is enough time to address negative emotions arising from the group experience. As such, this exercise should be modified by adjusting it to the needs of group members or removing it entirely.

Participants also proposed that exercises for independent use should be sent to them every week instead of sending them all at once before the final meeting. This is a recommended procedure because it may be easier to apply exercises independently but discuss them in group meetings.

### Strengths and Limitations

The data were collected during different seasons, so participants’ experiences in darker seasons (autumn and winter) and different weather conditions were taken into consideration. Because FWN has a clear structure that can be repeated, it is moderately easy to bring it into use in the health care system and modify it for the general public as well as for use in education.

There were some shortcomings in the research. The participants, who volunteered, were mostly female. This may have affected the results in such a way that, for example, the group dynamics of the ‘women’s groups’ differed from the mixed or male groups. Since only few men were in any one group at the same time, some of the men may have felt as ‘not belonging’ and possibly received less social support relevant to their situation.

The differences in how often the different stages (Path stage in particular) were mentioned in the feedback were moderately small. This may have been due to the fact that the questions for feedback referred to the treatment as a whole, which included the group, nature and the exercises. So, the participants did not necessarily recognise different stages during or after a treatment. For example, if it had been asked how FWN could be utilised after the rehabilitation, descriptions of the Path stage would probably have being mentioned more frequently.

The data were collected mainly *via* survey (pen-and-pencil and/or e-mail, but some participants were interviewed by telephone because they had not replied to feedback questions). It seemed that the phone discussions enabled more versatile and longer answers. To provide the participants with similar and impartial treatment, it would be better to have a feedback discussion with everybody by telephone in future studies. Participants also returned their feedback forms to the group facilitator, so some participants may have been reluctant to give critical feedback directly in this way. Further, it would be important to collect feedback from those participants who dropped out from the FWN group and compare them with those who stayed for the whole treatment.

The restrictions caused by the Covid pandemic interrupted some of the groups and meetings were moved online. Some of the participants also dropped out because of Covid. The pandemic came to everybody as a surprise, so it was not possible to prepare for it by the spring of 2020. In future, it would be important to inform participants beforehand that some or all meetings might be carried out online so as to reduce possible disappointments and dropouts.

## Conclusion

In the present study, participants’ feedback on our new NBI for depression rehabilitation (FWN treatment) was qualitatively examined. The FWN treatment was based both on earlier research results in eco and environmental psychology (e.g., Salonen et al., 2018), psychotherapeutic theories and professional psychologists’ experiences in therapeutic work. The group-form intervention included holistic psychological methods that can be repeated and which can be developed and examined further in the future. The participants’ experiences of the key elements of the social support, nature environment and FWN exercises, which differed at three separate stages, were mainly positive. This supports the inclusion of these key components in future intervention versions. In future, FWN exercises should be developed further to better enable participants’ possibilities for concentration and being present in the moment (mindfulness), and exercises should be simplified to match the target group’s cognitive abilities.

## Data Availability Statement

The original contributions presented in the study are included in the article/supplementary material, and further inquiries can be directed to the corresponding author.

## Ethics Statement

The studies involving human participants were reviewed and approved by Ethical commission of the Tampere University Hospital. The patients/participants provided their written informed consent to participate in this study.

## Author Contributions

KS as corresponding author has made mainly analysis, interpretation of data and writing. All authors have made contributions to planning, designing, collecting the data, participated in writing and commenting the content and also approved the version to be submitted in the journal. KK supervised the research project and revised the content critically.

## Funding

Our study was funded by the Social Insurance Institution of Finland (Kela).

## Conflict of Interest

The authors declare that the research was conducted in the absence of any commercial or financial relationships that could be construed as a potential conflict of interest.

## Publisher’s Note

All claims expressed in this article are solely those of the authors and do not necessarily represent those of their affiliated organizations, or those of the publisher, the editors and the reviewers. Any product that may be evaluated in this article, or claim that may be made by its manufacturer, is not guaranteed or endorsed by the publisher.
